# Neurovascular Coupling: Scientometric Analysis of 30 Years Research (1996–2025)

**DOI:** 10.1002/brb3.71058

**Published:** 2025-11-11

**Authors:** Xuenan Lang, Xuhang Fan, Minheng Zhang, Hongwei Liu, Haiyan Wu, Xiaodong Hu, Xiaojuan Yang, Haixia Fan

**Affiliations:** ^1^ First Hospital of Shanxi Medical University Taiyuan Shanxi Province China; ^2^ The First People's Hospital of Jinzhong Yuci Shanxi Province China; ^3^ Taiyuan City Central Hospital Taiyuan Shanxi Province China

**Keywords:** Alzheimer's disease, cerebral blood flow, fMRI, neurodegenerative disorders, neuroinflammation, neurovascular coupling

## Abstract

**Background:**

Neurovascular coupling (NVC) is the functional mechanism that links brain neural activity with the dynamic regulation of local blood flow and oxygenation. In recent years, there has been an increasing academic attention to the role of NVC in its pathophysiology and the application of new technologies.

**Objective:**

This study aims to map the research landscape related to NVC through scientometric analysis.

**Methods:**

Publications from the past 30 years were retrieved from the Web of Science Core Collection (WoSCC) database. Data were analyzed using CiteSpace, VOSviewer, and the bibliometrix R package, including co‐citation and keyword co‐occurrence network analyses. Key metrics such as publication counts and citation frequencies were assessed to identify trends and collaboration patterns among countries, institutions, and authors.

**Results:**

Among the 2047 articles included in the study, United States has maintained a clear leading position. Meanwhile, the number of Chinese research participants has grown rapidly over the past decade. The most prolific authors were Professors Iadecola Costantino and Tarantini Stefano. The research findings of Professor Tarantini Stefano have been widely recognized by researchers in the field. Keyword analysis identified “cerebral blood flow,” “neuronal activity,” and “neurovascular coupling” as dominant terms, emphasizing the central role of brain function and imaging techniques such as fMRI, TCD, and optical imaging. The emergence of “fNIRS,” “resting‐state fMRI,” and “autoregulation” highlights the growing impact of noninvasive neuroimaging in studying brain–blood flow interactions. Cluster analysis revealed key research themes including functional connectivity, nitric oxide‐mediated vascular regulation, cerebral autoregulation, Alzheimer's disease metabolism, and CO_2_‐induced hemodynamic modulation.

**Conclusion:**

Over the past three decades, NVC has emerged as a key research focus, driven by interdisciplinary collaboration and advances in brain connectivity, dysfunction, and technology. In the future, integrating artificial intelligence, multi‐omics analysis, and high‐resolution imaging will further elucidate NVC mechanisms in health and disease, promoting interdisciplinary translation and breakthroughs in neuroscience and brain health.

## Graphical abstract

Bibliometric landscape of neurovascular coupling (1996–2025). This graphical abstract summarizes the bibliometric structure and intellectual evolution of the neurovascular coupling field. (A) Displays the number of publications and citations per year from 1996 to 2025. (B) Presents circular plot of international collaboration. (C) Presents Journal citation network. (D) Displays the co‐authorship network and temporal evolution. (E) Depicts the keywords co‐citation timeline, revealing major thematic clusters such as “functional connectivity,” “nitric oxide,” “cerebral autoregulation,” and “optical imaging.”

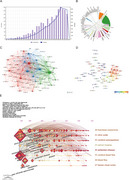

**Abbreviations**
AD: Alzheimer's disease
BBB: blood–brain barrier
CBF: cerebral blood flow
fMRI: Functional Magnetic Resonance Imaging
fNIRS: functional near‐infrared spectroscopy
HD: Huntington's disease
MCP: multiple‐country publications
NC: number of citations
NOS: Nitric oxide synthase
NP: number of publications
NVC: neurovascular coupling
NVU: Neurovascular Unit
OALM: Online Analysis Platform of Literature Metrology
PD: Parkinson's disease
PNAS: Proceedings of the National Academy of Sciences USA
SCP: single‐country publications
TCD: Transcranial Doppler
WoSCC: Web of Science Core Collection

## 1 Introduction

Formally introduced by National Institute of Neurological Disorders and Stroke in 2001, the neurovascular unit (NVU) is a functional complex of neurons, glial cells, vascular cells, and extracellular matrix that maintains brain homeostasis by coordinating neural activity with microcirculatory blood flow. Its core function, neurovascular coupling (NVC), ensures precise matching between neuronal activity and local perfusion (Attwell et al. 2010). Dysfunction of NVC has been increasingly implicated in the pathophysiology of neurodegenerative diseases, including Alzheimer's disease (AD) (Iadecola 2004; Sweeney et al. 2018; Zlokovic 2011), Parkinson's disease (PD) (van der Horn et al. 2024), Huntington's disease (HD) (Hu et al. 2025), etc. In these conditions, impaired NVC leads to disrupted cerebral blood flow regulation, contributing to metabolic stress, accumulation of toxic proteins, and neuronal injury. For instance, in AD, amyloid‐β deposition adversely affected endothelial function and pericyte signaling, thereby compromising the NVU's ability to match blood flow to neural demand (Kisler et al. 2017). These findings underscored the critical role of NVC not only as a biomarker for early diagnosis but also as a potential therapeutic target for mitigating disease progression in neurodegenerative disorders.

Bibliometric analysis, applied in fields like stroke and anesthesiology, offers an objective view of trends and frontiers by providing qualitative and quantitative insights into research (Alsbrook et al. 2023; Lee and Funk 2023; Renú et al. 2017). It uses knowledge mapping and clustering visualization to identify emerging associations and intersections, such as between metabolomics and cerebral small vessel disease (C. Chen and Song 2019; Y. Chen et al. 2021; Kendale et al. 2018). Bibliometrics quantitatively assesses scientific knowledge through citation data, acting as a performance evaluation. Combined with systematic mapping, it forms scientometric analysis, which classifies and maps scientific knowledge.

Despite significant progress in research on NVC, many key mechanisms remain to be fully explored. Future research needs to further investigate these mechanisms to better understand the role of the NVU in health and disease, and to provide a theoretical basis for the development of new therapeutic strategies (Kisler et al. 2017; Sweeney et al. 2018; Zlokovic 2008). Our study aims to fill this gap. Our main goal is to assess how research on NVC has evolved over the past 30 years. We will use co‐citation network analysis to pinpoint key studies and emerging trends. In addition, we plan to map out the research landscape by looking at contributions from different countries, institutions, authors, and journals. Research on NVC has significantly pushed forward the fields of neuroscience and clinical medicine, particularly in diagnosing, treating, and rehabilitating brain dysfunction. As metabolic imaging techniques improve, future research might combine changes in cerebral blood flow with metabolic dynamics, using multimodal imaging techniques. This could give us a fresh look at NVC and offer new ways to diagnose and treat neurological diseases early on.

## 2 Methods

### 2.1 Search Strategy and Data Collection

**FIGURE 1:**
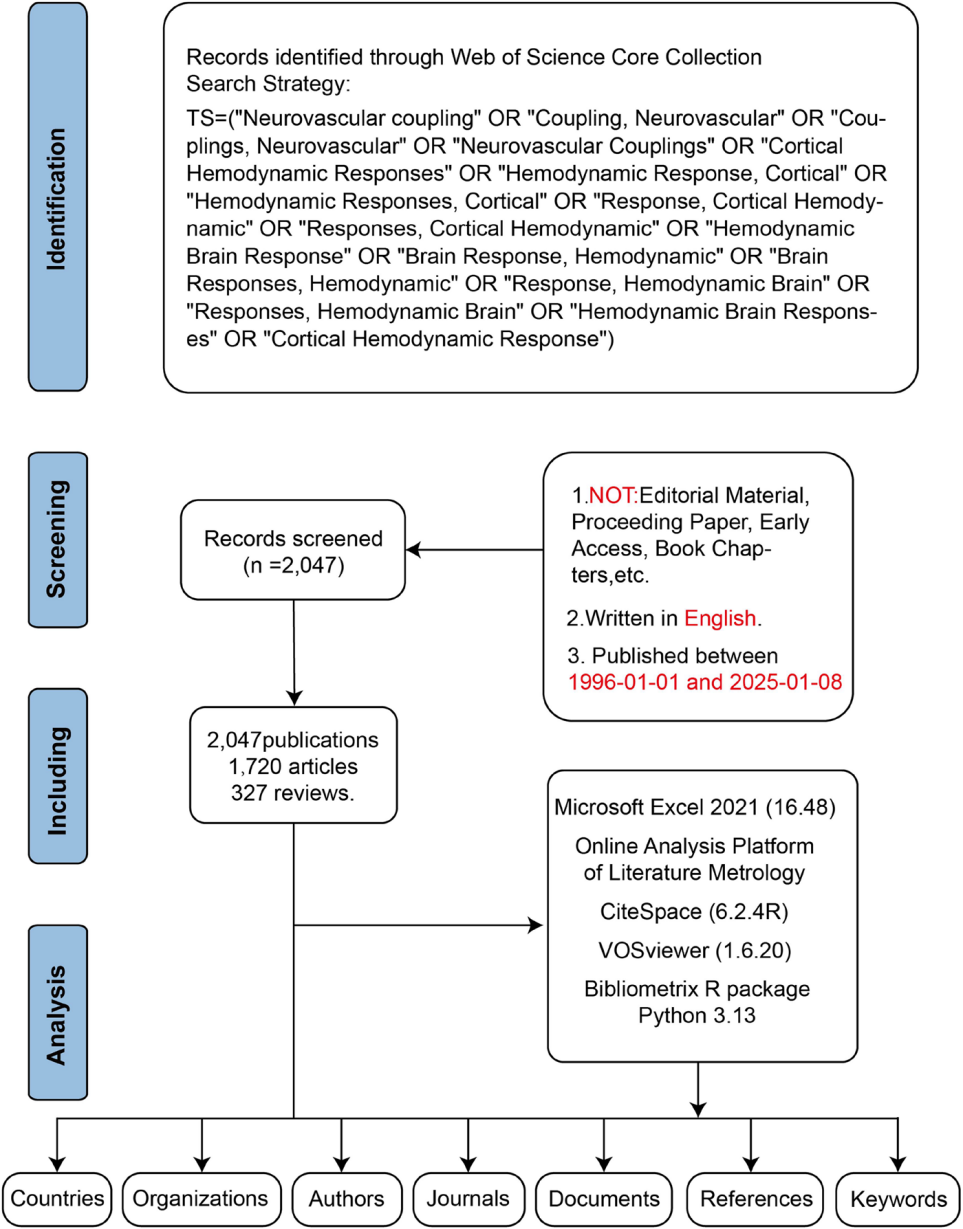
The flowchart for literature search, selection and analysis. Abbreviations: TS, topic search.

We selected Web of Science Core Collection (WoSCC) as our datasets. The following search strategy was employed: TS = (“Neurovascular coupling” OR “Coupling, Neurovascular” OR “Couplings, Neurovascular” OR “Neurovascular Couplings” OR “Cortical Hemodynamic Responses” OR “Hemodynamic Response, Cortical” OR “Hemodynamic Responses, Cortical” OR “Response, Cortical Hemodynamic” OR “Responses, Cortical Hemodynamic” OR “Hemodynamic Brain Response” OR “Brain Response, Hemodynamic” OR “Brain Responses, Hemodynamic” OR “Response, Hemodynamic Brain” OR “Responses, Hemodynamic Brain” OR “Hemodynamic Brain Responses” OR “Cortical Hemodynamic Response”). To minimize bias, we systematically retrieved articles and reviews published between January 1, 1996, and January 8, 2025, and downloaded them within one day (January 8, 2025). Exclusions were applied to remove proceeding papers, corrections, early access articles, news items, book chapters, retractions, reprints, biographical items, book reviews, meeting abstracts, editorial materials, and letters. Only articles and reviews in English were retained. Ultimately, a total of 2047 studies (Figure 1).

### 2.2 Data Processing

The data covered the period from January 1, 1996 to January 8, 2025, with the data segmented into one‐year time slices for detailed analysis. All records from the WoSCC were exported as “full records and cited references” in plain text format and Tab format14. Initial data was conducted using Microsoft Excel 2021 (version 16.48) and Online Analysis Platform of Literature Metrology (OALM) (http://bibliometric.com/). Python 3.13 was utilized to analyze annual publication trends, total publications per year, and the fitted trend curve. CiteSpace (6.2.4R, 64‐bit Advanced Edition) was employed to generate knowledge maps and cluster analysis of institutions, documents, references, and keywords. In addition, VOSviewer (1.6.20) applied full counting to produce visual representations for authors, journals, and collaborative network analysis15. The bibliometrix R package was used to perform historiographic analysis by tracking trends in journals and authors, as well as calculating key metrics such as the *g*‐index, *h*‐index, number of citations (NC), and number of publications (NP) (Figure 1).

## 3 Results

### 3.1 Trends and Academic Contributions in Publications

**FIGURE 2:**
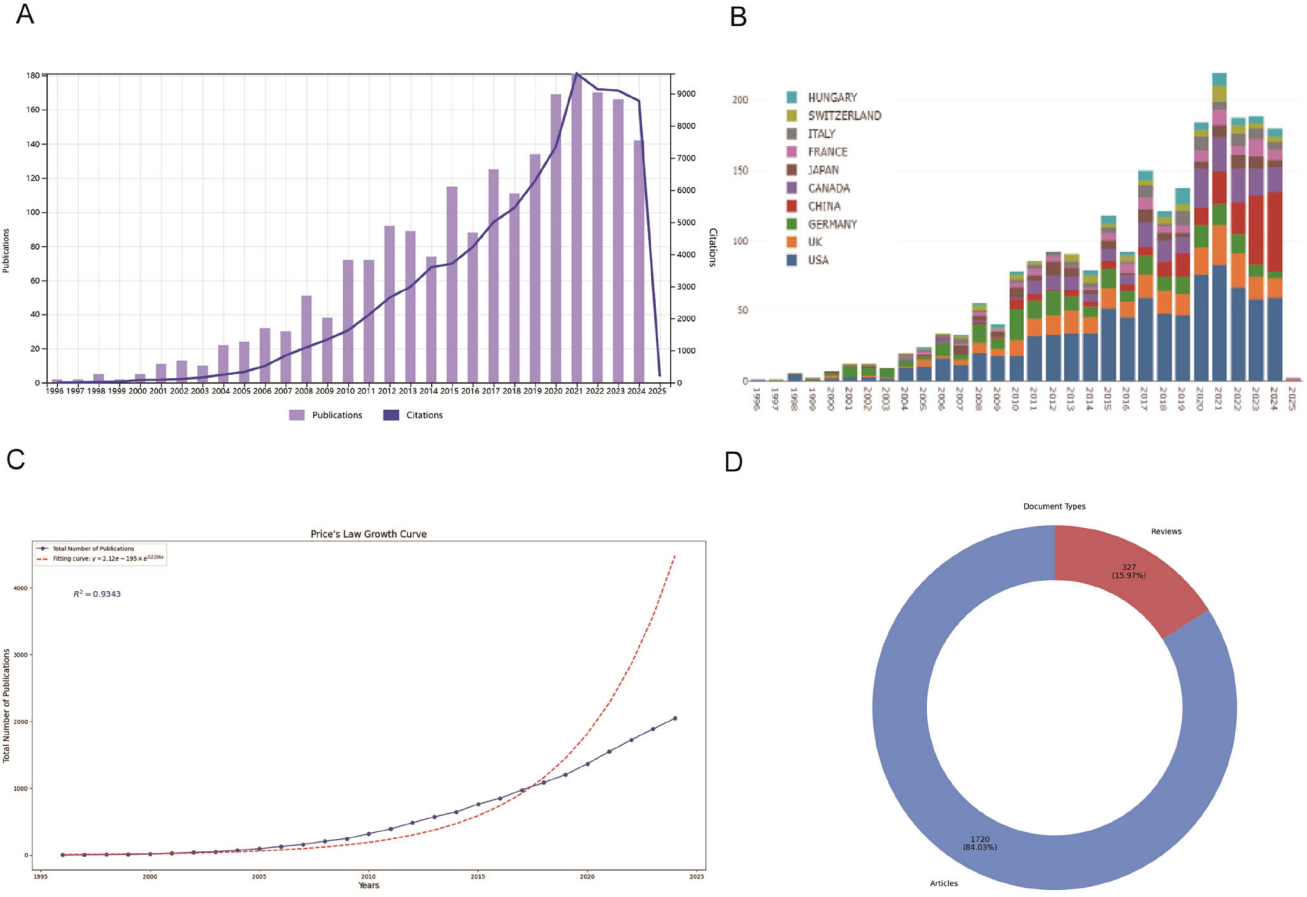
**Overview of publications and citations on neurovascular coupling (1996–2025). (A)** The number of publications and citations per year from 1996 to 2025. The bar graph (purple) represents the number of publications each year, while the line graph (blue) represents the corresponding citations. **(B)** A stacked bar graph showing the number of publications by country from 1996 to 2025, with each country represented by a different color. **(C)** Price's Law Growth Curve. **(D)** Distribution of document types.

Figure 2A,B illustrate the annual NP and corresponding citations from 1996 to 2025. The publication count has risen steadily over the years, reaching over 200 by 2022. In contrast, the NC experienced a substantial surge starting in the early 2000s, peaking at nearly 10,000 in 2022 and 2023. These documents exhibit a remarkable annual growth rate of 16.5%, reflecting a robust increase in scholarly output. The notable decline in the volume of documents in 2025 is likely due to the fact that the data only included records up to January 19, 2025. As of the retrieval date, a total of 2047 papers sourced from various sources were published over the past three decades. Among them, articles form the majority, with 1720 articles (84.3%) and 327 reviews (15.7%) (Figure 2D, Table S1). In addition, Figure 2C shows the cumulative NP over the study period, revealing an exponential growth trend that aligns with the general rise in scientific productivity. To further understand this growth, Price's Law was applied to fit an exponential growth model (Figure 2C), yielding a high correlation coefficient (*R* ^2^ = 0.9343), confirming publication trends' exponential nature.

### 3.2 Analysis of Cooperation Between Countries

**FIGURE 3:**
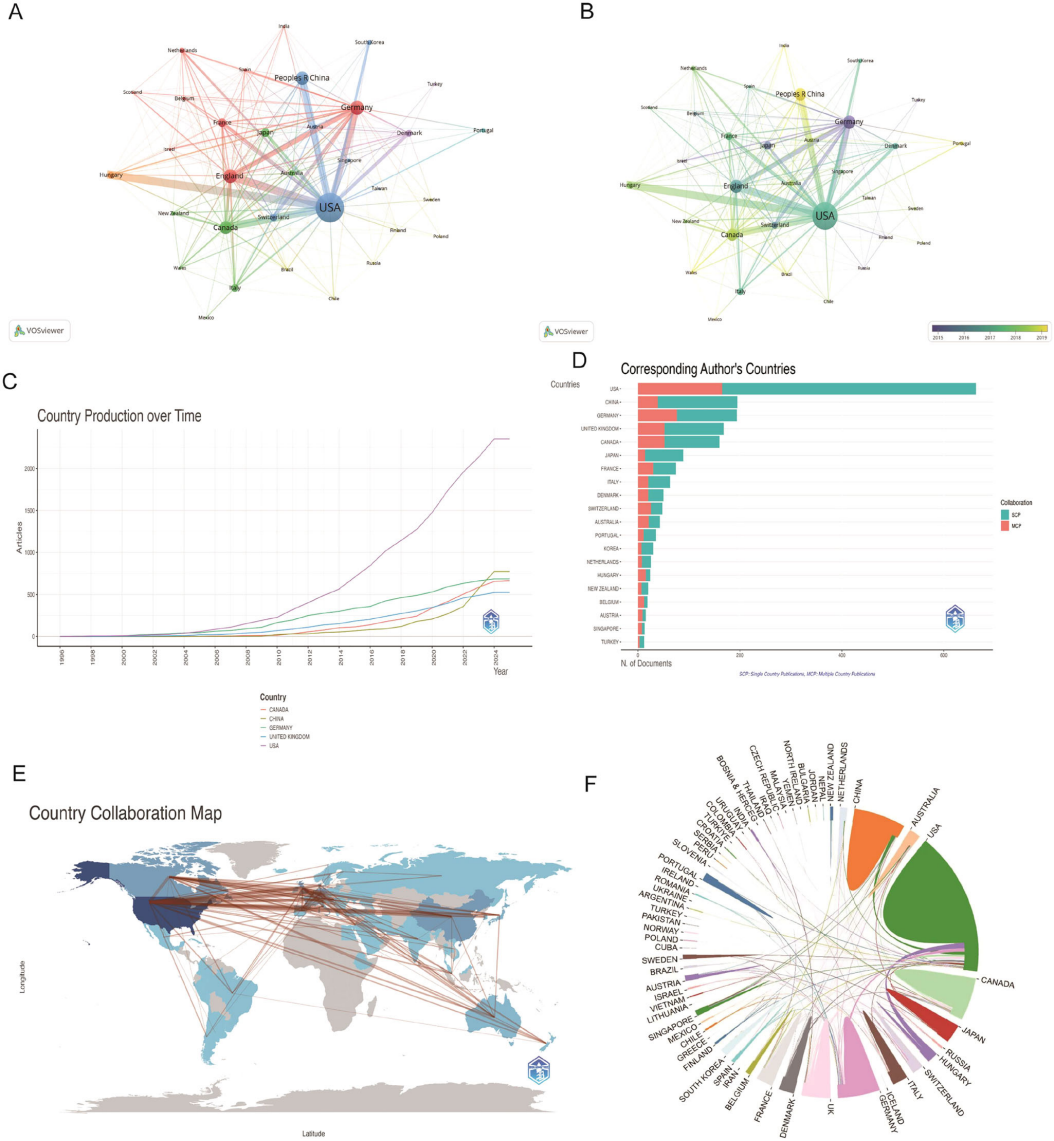
**Global trends and collaboration networks in scientific publications on neurovascular coupling. (A, B)** Visual representation of country‐based scientific collaborations. Nodes represent countries, with node size reflecting the number of collaborations. Edges between countries represent the intensity and frequency of collaborations. (A) Shows the network, while (B) illustrates the temporal evolution of collaboration intensity, with colors indicating the time span, from earlier years to more recent ones. **(C)** Country publication trends. The *x*‐axis spans years, and the *y*‐axis shows citation counts. **(D)** The top 10 countries responsible for the number of studies **(E)** Global collaboration map of co‐authorship networks. Darker blue shades reflect higher publication output, with lines depicting international collaborative ties. **(F)** Circular plot of international collaboration. Abbreviations: MCP, multiple‐country publications; OALM, Online Analysis Platform of Literature Metrology; SCP, single‐country publications.

The country collaboration map (Figure 3A,B) illustrates strong international partnerships, particularly between the U.S., Europe, and Asia. The United States leads in academic output, contributing 663 articles (32.4%) from 1996 to 2025—far exceeding other nations like China and Germany, which produced 195 (9.5%) and 194 (9.5%) articles, respectively (Table S2). Figure 3C presents the article production trends for China, Germany, Canada, the UK, and the U.S., which are the top five countries with the highest NP. The U.S. shows steady growth and maintains its leading position, while China has experienced significant growth, particularly from the early 2010s, positioning itself as a major player in academic publishing. Germany also shows growth but still lags behind China's output. There is also a notable trend toward increased multiple‐country collaborations, indicating a shift toward more globalized research efforts (Figure 3A,B,E,F). Figure 3D shows the annual publication count for each country, further reaffirming the leading position of the U.S. Figure 3D distinguishes between single‐country publications (SCP) and multiple‐country publications (MCP). The U.S. dominates in both SCP and MCP, showcasing its strength in both independent research and international collaborations. China follows closely, particularly in SCP, highlighting its growing influence in the academic sphere (Table S2). Overall, these findings highlight the importance of international collaboration in enhancing research visibility and impact, while also reflecting the evolving dynamics of global scholarship in recent decades.

### 3.3 Analysis of Cooperation Between Institutions

**FIGURE 4:**
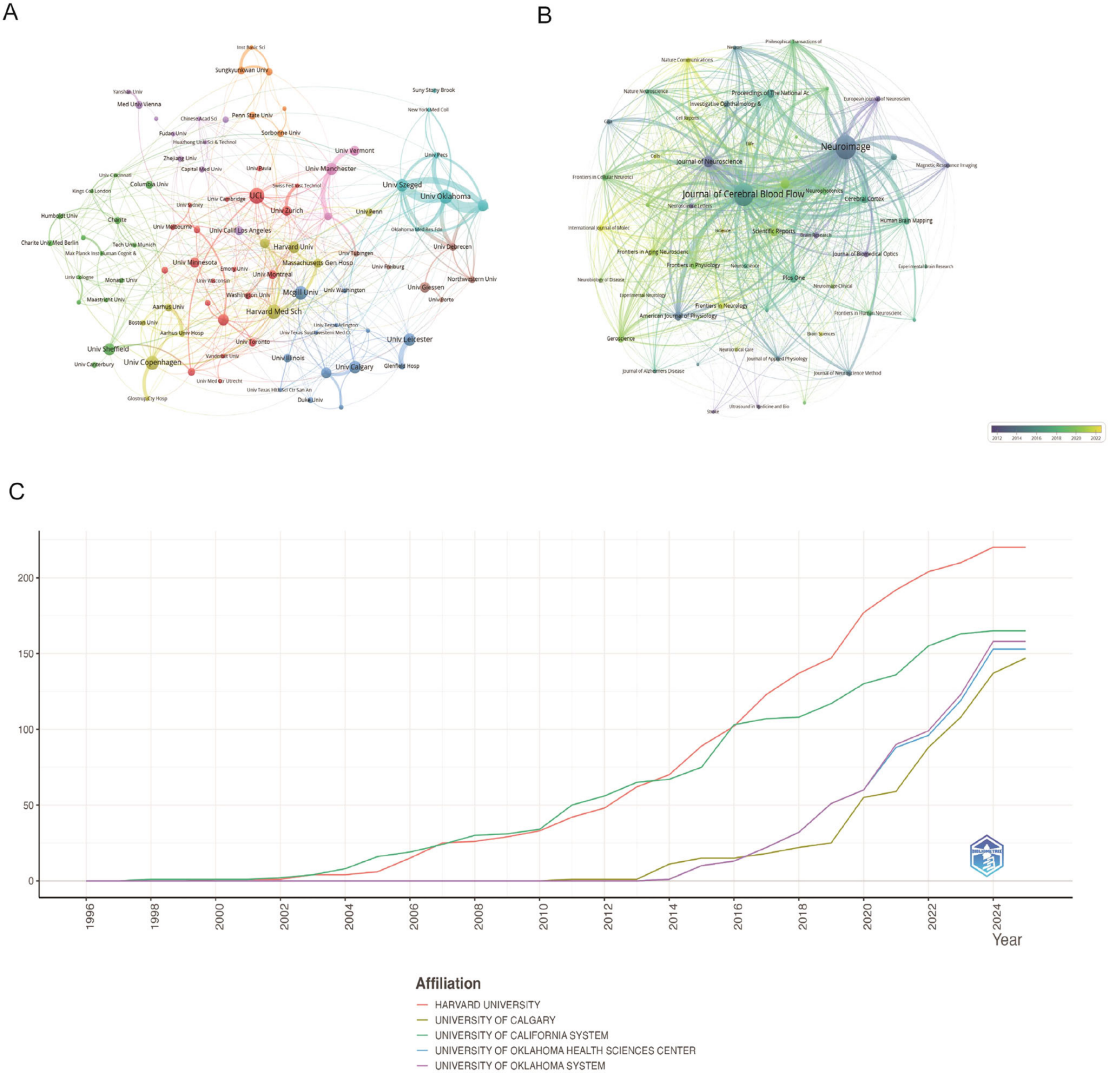
**Institutional contributions and collaboration networks on neurovascular coupling. (A)** Institutional collaboration network. Node size corresponds to collaboration extent, and colors denote distinct regional or institutional clusters. **(B)** Shows the distribution of collaboration over time, from earlier to more recent years. **(C)** Institutional publication trends. The *x*‐axis denotes years, and the *y*‐axis indicates publication counts.

Utilizing bibliometrix package analysis, 484 academic institutions across 27 nations were identified. Harvard University emerged as the most prolific contributor with 220 publications, followed by University of California System (165 papers), University of Oklahoma System (158 papers), University of Oklahoma Health Sciences Center (153 papers), and University of Calgary (143 papers) (Table S3). Network mapping of academic cooperation highlighted universities as primary contributors (Figure 4A,B), featuring Harvard University as the central node. The strongest collaborative ties were identified between University of Oklahoma System and its affiliated Health Sciences Center, reinforcing the observed connection between institutional alliances and geographic adjacency. Figure 4C showed that while Harvard University and the University of California System have consistently held prominent rankings, notable expansion has been observed in the University of Oklahoma System and University of Calgary's scholarly productivity since 2023, with both institutions surpassing Harvard's publication output in 2023 and 2024 respectively.

### 3.4 Authors and Co‐Cited Authors

**FIGURE 5:**
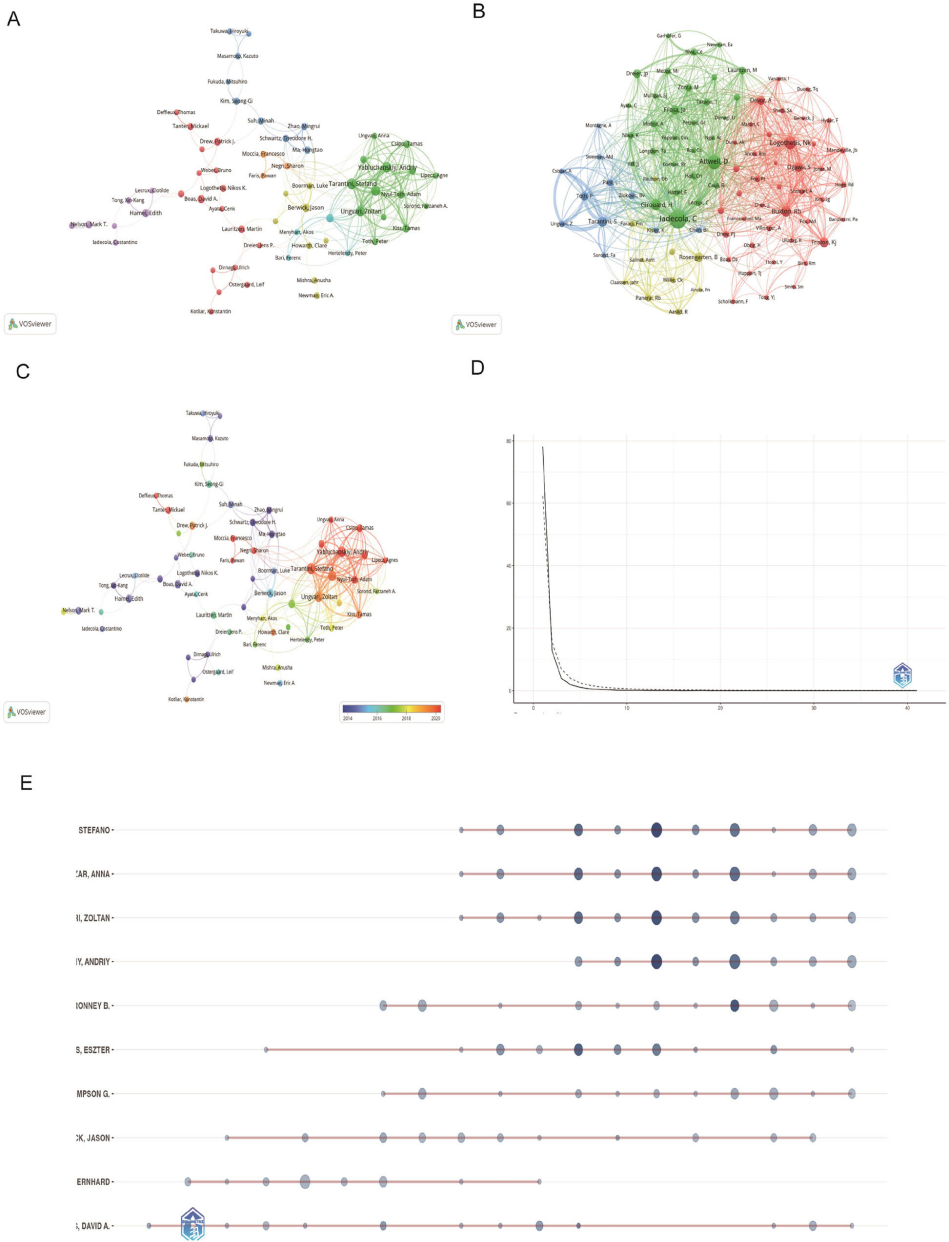
**Co‐authorship and co‐cited authorship networks on neurovascular coupling. (A)** Co‐authorship network: Nodes represent authors, sized by publication count. Colors indicate collaboration clusters. **(B)** Co‐cited authorship network: Nodes, connected by collaborative publications, have edge thickness proportional to joint works. Clusters, marked by colors, reveal research groups. **(C)** Co‐authorship network and temporal evolution. **(D)** Lotka's law fitting curve: Highlights the distribution of productivity and collaboration patterns among authors. **(E)** Author collaboration over time: A horizontal bar chart displaying the collaboration timeline of specific authors. Each bar represents an author, with deeper colors indicating more‐significant contributions.

Our analysis revealed contributions from 8494 scholars over the 28‐year study period (Table S1). Stefano Tarantini emerged as the most productive researcher with 41 publications. Subsequent rankings showed Anna Csiszar (38 papers), Zoltan Ungvari (37 papers), Andriy Yabluchanskiy (36 papers), and Ronney B Panerai (30 papers) completing the top five contributors (Figure 5A,B). As shown in Table 1, scholarly impact evaluations using *h*‐index, *g*‐index, and *m*‐index metrics identified Tarantini (*h*‐index: 29, total citations: 2584) and Csiszar (*h*‐index: 27, citations: 2488) as the most influential researchers, both commencing their significant contributions in 2014 (Figure 5C). Analysis of 2047 referenced works identified Costantino Iadecola as the most frequently cited scholar (2838 citations). The citation leaderboard further included Tarantini, Ungvari, Csiszar, and David A. Boas in the top five positions (Figure 5E), demonstrating their substantial academic impact in NVC studies. Author productivity distribution followed the generalized Lotka's law framework, expressed as *y* = c/*x*ⁿ, where *y* represents authors publishing *x* papers, and c and n are constants. Regression analysis yielded the function *y* = 5661.9*x* ^−3.635^ with strong model fit (*R* ^2^ = 0.9343), confirming adherence to this bibliometric principle (Figure 5D). This inverse power relationship indicates that most researchers (5661.9 times the reciprocal of *x* ^3.635^) contributed to single publications. Collaboration network analysis demonstrated three key patterns: (1) High‐output authors formed intensive cooperative clusters, frequently aligned with institutional affiliations, (2) major research clusters emerged around cerebral hemodynamics, Functional Magnetic Resonance Imaging (fMRI) applications, and neurovascular pathologies, and (3) limited inter‐cluster collaboration was observed despite intra‐group cooperation (Figure 5A,C). Distinct investigative subgroups focused on specialized domains including cerebral autoregulation mechanisms, resting‐state brain imaging techniques, and neurovascular pathophysiology in glaucoma and AD.

**TABLE 1:** Top 10 authors on neurovascular coupling research (1996–2025).

Rank	Author	h_index	g_index	m_index	TC	NP	PY_start	Articles	Articles fractionalized
1	Tarantini Stefano	29	41	2.417	2584	41	2014	41	3.6
2	Ungvari Zoltan	28	37	2.333	2552	37	2014	37	3.13
3	Csiszar Anna	27	38	2.25	2488	38	2014	38	3.16
4	Yabluchanskiy Andriy	24	36	2.667	1773	36	2017	36	2.86
5	Farkas Eszter	20	25	1.176	1996	25	2009	25	2.65
6	Hamel Edith	18	20	0.9	1362	20	2006	20	4.46
7	Kiss Tamas	18	19	2	1308	19	2017	19	1.48
8	Nelson Mark T.	17	18	0.85	1422	18	2006	18	5.05
9	Boas David A.	16	20	0.8	2162	20	2006	20	2.6
10	Balasubramanian Priya	15	18	2.143	825	18	2019	18	1.38

Abbreviations: NP, number of publications; PY_start, year of first publication; TC, total citations.

### 3.5 Journal Analysis

**FIGURE 6:**
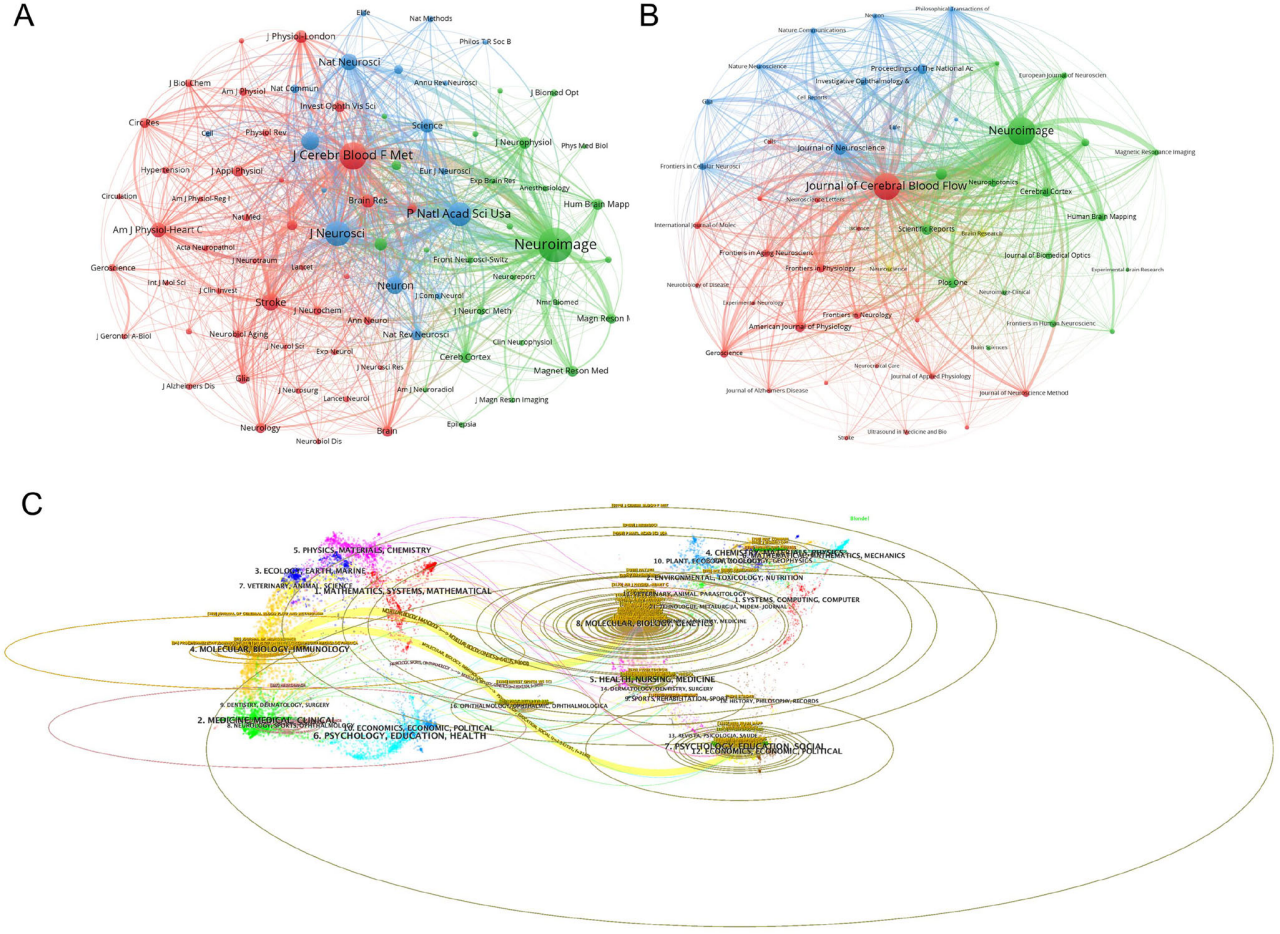
**Comprehensive analysis of journals and citation networks related to neurovascular coupling. (A)** Journal citation network: Nodes represent journals, connected by citations. Colors (green, blue, red) indicate thematic clusters of frequent inter‐citation. **(B)** Journal co‐citation network: Nodes, sized by co‐citation frequency, are linked by co‐citation ties. Colors denote clusters of commonly co‐cited journals. **(C)** Dual‐map overlay analysis: The left side with different colors represents different types of cited journals, while the right side with different colors represents different types of citing journals.

Our analysis using the bibliometrix package was performed on 2047 papers published across 484 journals (Table 2). ***NeuroImage*** topped the list with 187 publications, followed by ***Journal of Cerebral Blood Flow and Metabolism*** (169 papers), ***Journal of Neuroscience*** (52 papers), ***Proceedings of the National Academy of Sciences of the USA*** (34 papers), ***American Journal of Physiology‐Heart and Circulatory Physiology*** (34 papers), and ***Cerebral Cortex*** (33 papers) (Figure 6B,D; Table 2). These journals saw a significant increase in publication volume after 2006 (Figure 6D), which aligns with the identified developmental phases in the publication timeline. In terms of the *h*‐index, ***NeuroImage*** had the highest influence in this research domain (*h*‐index: 56). Other top journals by *h*‐index included ***Journal of Cerebral Blood Flow and Metabolism*** (*h*‐index: **49**) and ***Proceedings of the National Academy of Sciences of the USA*** (h‐index: **38**).

**TABLE 2:** Top 10 influential journals on neurovascular coupling research (1996–2025).

Rank	Source	h_index	g_index	m_index	TC	NP	PY_start	PP	IF	JCR
1	Neuroimage	56	84	2.24	8686	187	2001	9.13%	5.3	1
2	Journal of Cerebral Blood Flow and Metabolism	49	86	1.815	8499	169	1999	8.25%	6.3	1
3	Journal of Neuroscience	38	52	1.727	5485	52	2004	2.54%	5.3	2
4	Proceedings of the National Academy of Sciences of the USA	26	34	1.238	2909	34	2005	1.66%	11.1	1
5	American Journal of Physiology‐Heart and Circulatory Physiology	22	34	0.815	1925	34	1999	1.66%	4.8	2
6	Cerebral Cortex	20	33	0.952	1707	33	2005	1.61%	5.2	1
7	Geroscience	20	24	2.222	1143	24	2017	1.17%	5.3	1
8	Plos One	17	31	1.133	982	34	2011	1.66%	3.7	2
9	Neuron	16	30	1.223	958	33	2013	1.32%	4	2
10	Nat Neurosci	15	29	1.589	890	30	2002	1.27%	5.2	1

Abbreviations: IF, impact factor; JCR, journal citation reports.

Dual‐map overlay analysis using CiteSpace (Figure 6C) revealed citation patterns between citing and cited journals. The left side of the map represents citing journals, which are mainly clustered in “Molecular Biology, Immunology, Physics, and Psychology,” while the right side shows cited journals. Tightly interconnected clusters indicate strong co‐occurrence relationships, with major clusters including core journals and disciplines. Key research hotspots and long‐standing priorities were identified in fields such as healthcare medicine, psychology, molecular biology, and immunology.

### 3.6 Bibliographic and Citation Analysis

**FIGURE 7:**
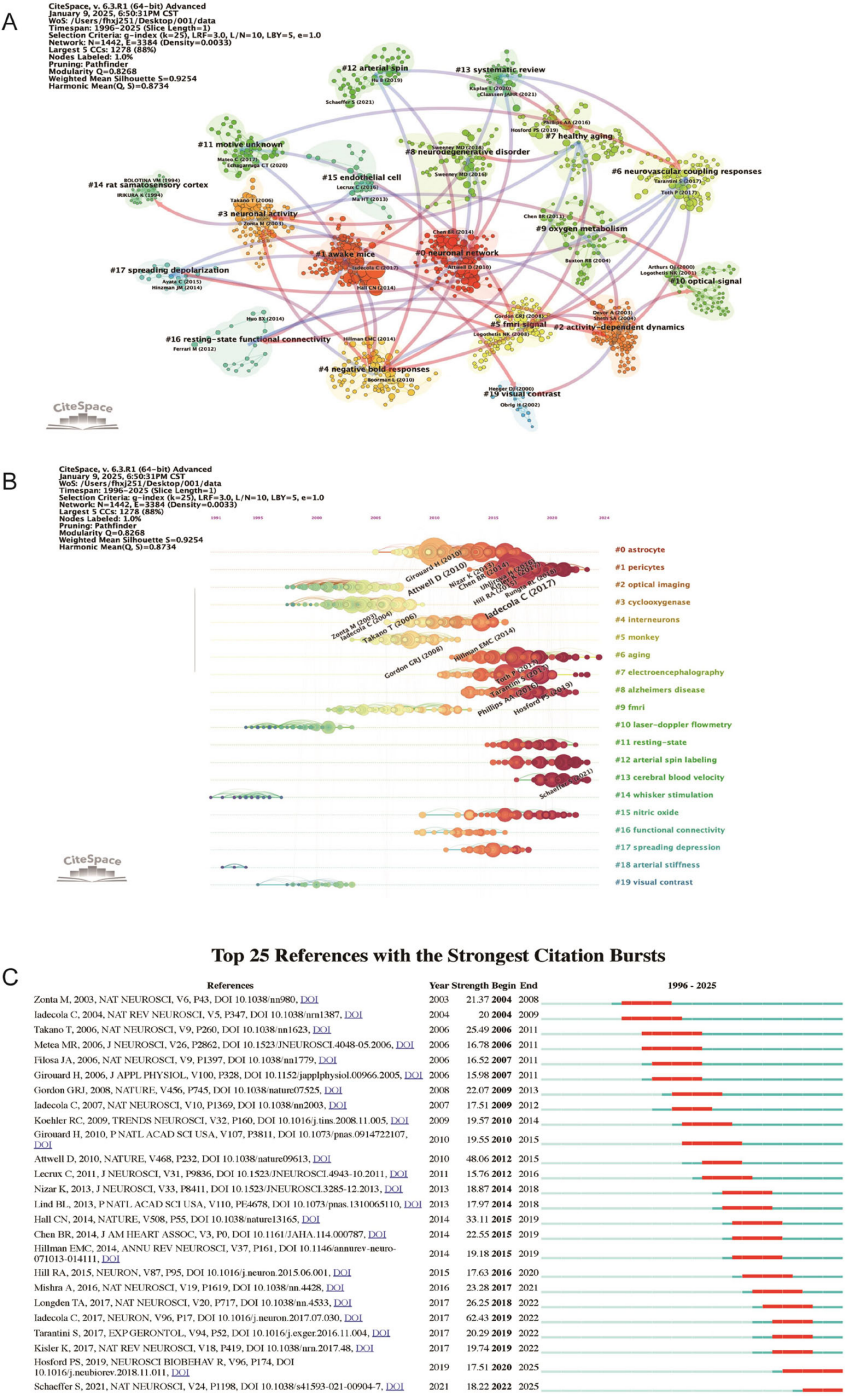
**Network visualization of references on neurovascular coupling. (A)** Clustered co‐citation network: Nodes, sized by citation frequency, are grouped by modularity with keyword labels for major clusters. Edge thickness reflects co‐citation strength; colors highlight thematic diversity. **(B)** Timeline clustering of publications **(C)** Top 25 References with strongest citation bursts on neurovascular coupling research. The burst timeline shows the periods during which these bursts occurred, with red segments indicating the time intervals when each reference received heightened attention.

To evaluate the impact of the 2047 papers included in this study, we utilized the CiteSpace software package to analyze the citation patterns of the most frequently cited papers both locally and globally. The paper “Iadecola C, 2017, Neuron” ranked first in terms of cross‐references, with a total of 264 mentions (Figure 7A,B). On a global scale, the same paper “Iadecola C 2017, Neuron” (Iadecola 2017) also emerged as the most cited, accumulating a total of 1396 citations (Table 3). The fact that “Iadecola C 2017, Neuron” (Hall et al. 2014) topped both local and global rankings underscores its significant influence within the global scientific community and this particular field. This paper primarily investigates the relationship between neurodegenerative diseases, particularly AD, and cerebrovascular diseases. The author suggests that cerebrovascular diseases might accelerate the progression of neurodegenerative diseases. It highlights the critical role of the cerebrovascular system in neurodegenerative diseases, especially how blood–brain barrier (BBB) dysfunction can contribute to neurodegenerative changes. By integrating research from neuroscience and vascular biology, the paper presents a novel perspective, suggesting a complex interplay between cerebrovascular diseases and neurodegenerative diseases. It calls for future research to focus on the intersection of these two fields to develop new therapeutic strategies.

**TABLE 3:** Top 25 most cited publications based on bibliometrix analysis (1996–2025).

Rank	Author	Year	Journal	Paper	DOI	Total citations	TC per year	Normalized TC
1	Iadecola C	2017	Neuron	The Neurovascular Unit Coming of Age: A Journey through Neurovascular Coupling in Health and Disease	10.1016/j.neuron.2017.07.030	1396	155.11	23.48
2	Haydon PG	2006	Physiol Rev	Astrocyte Control of Synaptic Transmission and Neurovascular Coupling	10.1152/physrev.00049.2005	983	49.15	5.49
3	Girouard H	2006	J Appl Physiol	Neurovascular coupling in the normal brain and in hypertension, stroke, and Alzheimer disease	10.1152/japplphysiol.00966.2005	927	46.35	5.18
4	Iadecola C	2007	Nat Neurosci	Glial regulation of the cerebral microvasculature	10.1038/nn2003	852	44.84	8.4
5	Kisler K	2017	Nat Rev Neurosci	Cerebral blood flow regulation and neurovascular dysfunction in Alzheimer disease	10.1038/nrn.2017.48	801	89	13.47
6	Ohab JJ	2006	J Neurosci	A Neurovascular Niche for Neurogenesis after Stroke	10.1523/JNEUROSCI.4323‐06.2006	705	35.25	3.94
7	Obrig H	2003	J Cerebr Blood F Met	Beyond the Visible—Imaging the Human Brain with Light	10.1097/01.WCB.0000043472.45775.29	676	29.39	3.71
8	D'esposito M	2003	Nat Rev Neurosci	Alterations in the BOLD fMRI signal with ageing and disease: a challenge for neuroimaging	10.1038/nrn1246	646	28.09	3.55
9	Maslov K	2008	Opt Lett	Optical‐resolution photoacoustic microscopy for in vivo imaging of single capillaries	10.1364/OL.33.000929	629	34.94	7.65
10	Burda JE	2016	Exp Neurol	Astrocyte roles in traumatic brain injury	10.1016/j.expneurol.2015.03.020	538	53.8	9.55

Abbreviation: DOI, digital object identifier.

Using CiteSpace, we generated a timeline‐based clustering analysis of the literature (Figure 7B). High‐frequency keywords were spread across core biological themes, such as astrocytes (star‐shaped glial cells), interneurons, and pericytes, indicating that the NVU is a current research hotspot. In terms of disease mechanisms, the link between AD and spreading depression shows that electrophysiological abnormalities in neurodegenerative diseases are a hot topic. In the realm of monitoring technologies, laser‐doppler flowmetry and arterial spin labeling appeared frequently, suggesting that cerebrovascular hemodynamics research is a technology‐driven field. In the area of brain functional imaging, fMRI, resting‐state, and functional connectivity highlight the dominant role of neuroimaging in brain network research. Emerging keywords and frontier directions, such as the combination of arterial stiffness and aging, point to the contribution of vascular aging to neurodegenerative diseases. Nitric oxide, as a signaling molecule, is anticipated to become a breakthrough in the study of NVC mechanisms in the future.

Following the evolution of the literature and the development of the discipline: During the technology‐driven period, traditional techniques like optical imaging and laser‐doppler were predominant. As the field entered the disease association period, clinical issues such as AD and aging became more prominent, reflecting a shift in research towards application. In future research directions, NVC is expected to break through technological bottlenecks: The development of high spatiotemporal resolution arterial spin labeling algorithms will enhance the quantitative accuracy of cerebral blood flow monitoring.

Using CiteSpace, a citation burst history chart was generated, listing the 25 references with the strongest citation bursts from 1996 to 2025 (Figure 7C). The 25 papers with the highest citation burst strength cover themes such as neuroscience, CBF, and NVC. The higher the burst strength value, the more significant the increase in citations within a specific time period (the highest was 62.43, from Iadecola C 2017) (Iadecola 2017). The burst duration is mostly 4–5 years, with recent bursts extending to 2025 (for example, papers from 2021 have bursts lasting until 2025). Early burst papers were from 2003 to 2010, with representative studies such as Ona M (2003, astrocyte regulation of blood flow) and Iadecola C (2004, NVC mechanisms). These focused on basic mechanism exploration. During the mid‐peak period (2010–2017), key papers included Attwell D (Attwell et al. 2010, NVC, IF: 48.06) (Attwell et al. 2010) and Hall CN (2014, capillary function, strength 33.11) (Hall et al. 2014). The significant increase in the burst strength reflects the technological breakthroughs (such as two‐photon microscopy) that have propelled the field forward. In the recent period (2017–2025): Representative papers include Iadecola C (2017, neurovascular diseases, strength 62.43) (Iadecola 2017) and Mishra A (2016, neuro‐metabolic regulation) (Masamoto et al. 2016) with the highest burst strength, indicating a shift in research focus towards disease mechanisms (such as AD and vascular aging). The core author is Iadecola C, with four highly burst papers (from 2004 to 2017), spanning the entire development of the neurovascular field. Attwell D and Hillman EMC had burst strengths of 48.06, making significant contributions to neuronal‐blood flow coupling and optical imaging techniques. The leading journals were Nature Neuroscience (8 papers), focusing on basic mechanisms, and Nature and Neuron, which published high‐burst clinical association studies.

Papers from 2010 to 2015, such as Hillman EMC (2014), had high burst strengths, corresponding to the popularization of two‐photon microscopy and fMRI technologies (Hillman 2014). In terms of animal model innovation, papers on non‐human primates (monkey) and transgenic mouse models have advanced disease mechanism research (Ungvari et al. 2017). After 2017, papers such as Tarantini S (2017) had significant burst strengths, pointing to the association between vascular aging (arterial stiffness) and AD (Schaeffer and Iadecola 2021). NVC burst papers span the entire period (from Zonta et al. 2003 to Schaeffer S, 2021). In the future, it will be necessary to integrate multi‐omics data and dynamic imaging technologies to decipher the spatiotemporal regulatory mechanisms of the NVU in diseases (Schaeffer and Iadecola 2021; Zonta et al. 2003).

### 3.7 Keyword Analysis

Our analysis generated 4761 Keywords Plus and 3842 author keywords to summarize research focuses and identify hotspots and trends in the field.

#### 3.7.1 Keyword Frequency

**FIGURE 8:**
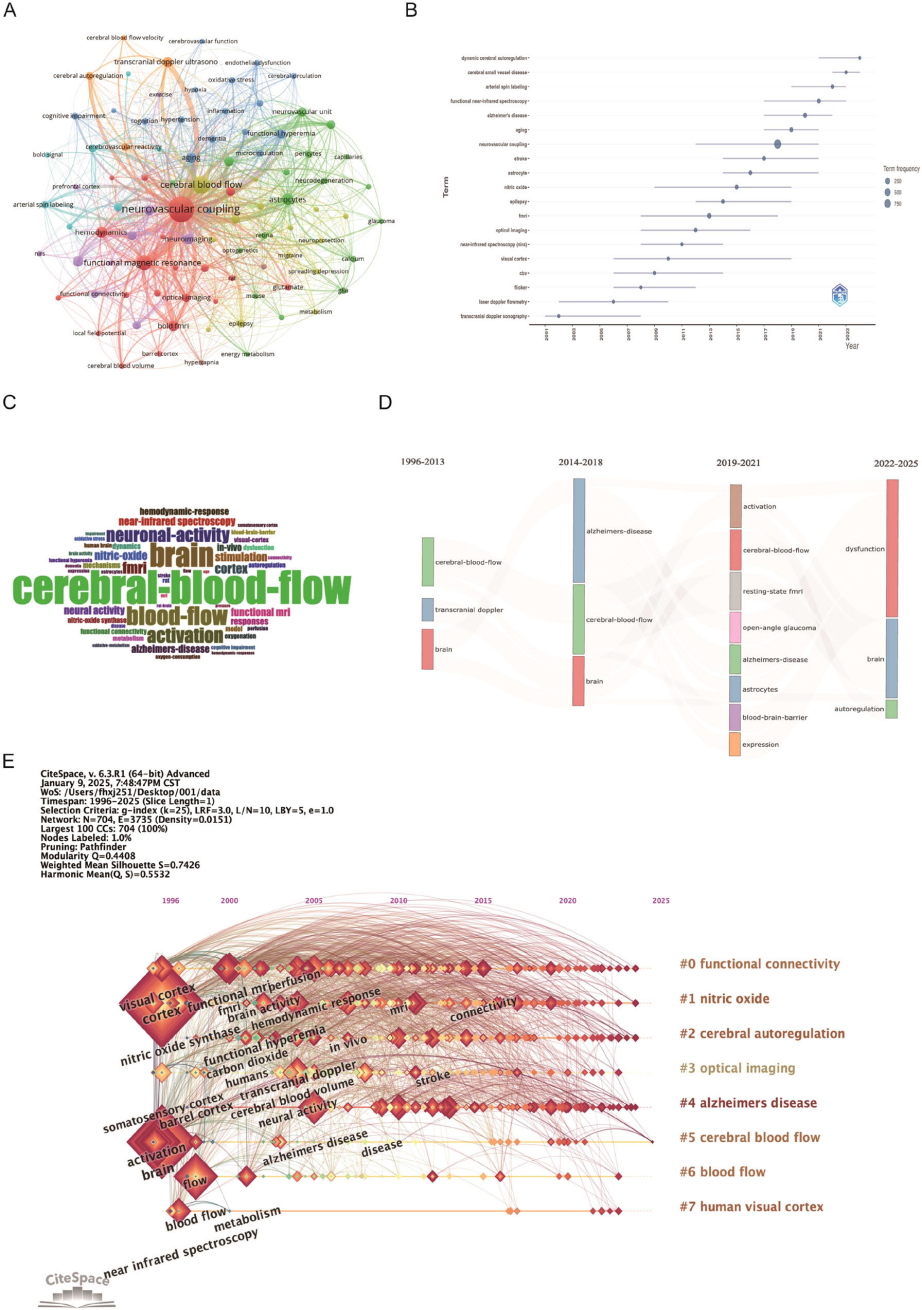
**Keywords mapping of neurovascular coupling. (A)** Keywords co‐occurrence network: Nodes represent keywords, sized by occurrence frequency (larger = more frequent), with edges indicating co‐occurrence and colors denoting thematic clusters. **(B)** Keywords timeline: Nodes, sized by frequency by cluster, illustrate the temporal evolution of research topics. **(C)** Author keywords word cloud: Word size reflects frequency. **(D)** Evolution of themes in Neurovascular Coupling research. **(E)** Co‐citation timeline: Clusters shown as horizontal lines, node size reflects co‐citation frequency, links indicate relationships, and node positions mark first co‐citation year.

Figure 8A presents 47 keywords, with high‐frequency terms including NVC (5 related terms): NVC, astrocytes, CBF, etc. *Imaging techniques* (6 related terms): Functional magnetic resonance, optical imaging, arterial spin labeling, etc. Low frequency but key themes in the figure include *Pericytes and microcirculation*, pericytes, capillaries, suggesting the need for more research on microvascular mechanisms in the future. *Metabolism‐inflammation intersection*: Energy metabolism, inflammation (both 3 times), a potential emerging direction. Besides, frequency analysis was conducted on Keywords Plus, author keywords, and all the keywords, and the results were presented in the form of a word cloud (Figure 8C). Excluding search terms, this word cloud illustrates the frequency and importance of terms related to cerebral blood flow research. The size of each word is proportional to its frequency and relevance in the study of brain activity and cerebral blood flow. Larger words, such as “CBF” and “brain,” indicate that these terms hold central positions in scientific literature, reflecting their widespread application in the research field. Other important terms, such as “neural activity,” “fMRI,” and “blood–brain barrier,” highlight key aspects related to brain function, metabolism, and technologies used to study CBF.

#### 3.7.2 Keyword Evolution

Figure 8B showed the evolution dynamics of keywords. *Dynamic cerebral autoregulation*: This term began to rise significantly in 2011 and peaked in the following years, indicating significant progress in exploring the mechanisms of cerebral blood flow regulation, especially in the prevention and treatment of stroke and other cerebrovascular diseases. The research areas involving “NVC” and “functional near‐infrared spectroscopy (fNIRS)” have experienced similar technological developments over the past decades, particularly in the study of the relationship between cerebral blood flow regulation and neural activity. The growth of these terms reflects the scientific community's increasingly profound understanding of the interaction between brain function and blood flow regulation, driven by the advancement of non‐invasive imaging technologies. Terms like “fMRI” and “fNIRS” represent significant breakthroughs in neuroimaging and brain function research, and their increasing usage frequency mirrors the development of these technologies. Over the past 20 years, researchers have paid increasing attention to the regulation of cerebral blood flow and its relationship with brain function. The popularity of terms such as “dynamic cerebral autoregulation” and “cerebral small vessel disease” reflects the growing importance of CBF regulation and small vessel diseases in neuroscience, demonstrating the progress of brain research on a more detailed and complex level (Meyer‐Baese et al. 2025).

Using the bibliometrix software package, the evolution of topics was analyzed, showing the changes in thematic terms within the field of brain and CBF research over four different time periods (1996–2013, 2014–2018, 2019–2021, 2022–2025). By analyzing the frequency of terms in different time periods, the gradual development of research in this field and the shift in academic focus can be observed. Each colored bar represents a key term or topic, and the size of the bar indicates the frequency of occurrence of that term in the corresponding period. Over time, the shift in topics is demonstrated by the movement of the colored bars. Terms such as “CBF,” “brain,” and “AD” persist across periods, while emerging terms like “activation,” “resting‐state fMRI,” and “autoregulation” appear in more recent studies. This timeline reflects the continuous development of the field, showing increasing attention to brain dysfunction, neurovascular regulation, and advanced imaging techniques in recent research (Claassen et al. 2021).

#### 3.7.3 Keyword Cluster Analysis

Cluster analysis of keywords using software algorithms can help us further understand the topics in this research field. In CiteSpace, cluster analysis was performed using the default algorithm, and clusters were labeled using the LLR algorithm, with a timeline map drawn (Figure 8E). The modularity *Q* value was 0.4408, indicating a well‐structured clustering network with significant results. The *S* value (average silhouette) was 0.7426, indicating high clustering efficiency and convincing classification results. The clusters were divided into 8 modules (Figure 8E), with more than 100 keywords in each of the 8 modules. The largest cluster was labeled CBF by the LLR algorithm, with the most frequent keywords being CBF (505 times), brain (361 times), and blood‐flow (282 times).

Cluster #0: Core keywords include *functional connectivity* (visual cortex, brain activity, neural activity). The main focus is on the dynamic coordination mechanisms between different brain regions, especially in visual information processing (e.g., the synergy between the visual cortex and other brain areas).

Cluster #1: Core keywords include *nitric oxide*, *nitric oxide synthase (NOS)*, and *hemodynamic response*. The research direction is the role of nitric oxide as a vasodilator in regulating CBF and NVC.

Cluster#2: Core keywords include *cerebral autoregulation*, *functional hyperemia*, and *cerebral blood volume*. The core mechanism is the stability of CBF in response to blood pressure fluctuations or changes in metabolic demand, involving the coordination of the NVU.

Cluster #4: Core keywords for *AD* include AD metabolism, blood flow, and spectroscopy. The research focus is on exploring early biomarkers of the disease, such as abnormal brain metabolism (decreased glucose utilization) and reduced blood flow (detected by PET or ASL‐MRI).

Cluster #5: Core keywords include *CBF*, *transcranial doppler (TCD)*, and *carbon dioxide (CO*_2_). The research content involves quantifying cerebral hemodynamic parameters (e.g., flow velocity, resistance index) and exploring the effects of CO_2_ on vascular tone (vasodilation induced by hypercapnia).

This clustering analysis reveals research threads across multiple scales (molecular, systems, clinical), providing a clear framework for future interdisciplinary collaboration. In future clinical translation, developing combined therapeutic strategies targeting the vascular regulation axis (clusters #1, #2, #5) could provide an empirical basis for functional connectivity, reflecting the potential for brain network dynamic modeling or the application of artificial intelligence in neuroscience.

## 4 Discussion

NVC is a core area in neuroscience and CBF research, involving the precise regulatory process of matching neuronal activity with local blood flow dynamics (Barloese et al. 2022; Claassen et al. 2021; Iadecola 2017; Metea et al. 2007). Through bibliometric analysis, this study has revealed the research trends, hotspots, and frontier directions in the field of NVC, providing important references for its future development.

The research output in the field of NVC has shown exponential growth over the past 30 years, with a particularly significant increase in research activities after 2020. The results of keyword and reference clustering analysis highlight the primary areas of interest for researchers. Based on the current findings and our understanding of the field, we suggest that several key research hotspots are emerging, including investigations into pathophysiological mechanisms, their interplay with neurodegenerative diseases, and the application of advanced multimodal imaging.

### 4.1 Bidirectional Regulatory Mechanisms: Pericyte Dynamics and Molecular Pathways

Traditionally, the central debate regarding NVC has revolved around the role of astrocytes’ “terminal feet” in transmitting neuronal demands to arteries via calcium signaling. In recent years, two‐photon imaging has shed substantial light on the role of pericytes in NVC, revealing that pericytes directly respond to neuronal activity by modulating local blood flow through precapillary sphincters. Notably, pericytes can respond in under 1 s, a speed that significantly outpaces that of arteries, which typically take around 10 s to react (Masamoto et al. 2016).

Studies have confirmed that the interaction between neurons and microvessels is bidirectional: Changes in the degree of neuronal excitation (or inhibition) cause changes in capillary blood flow and oxygenation, and changes in cerebral capillary blood flow and oxygenation can also lead to irreversible neuronal damage (Attwell and Iadecola 2002). The molecular mechanisms underlying NVC are primarily driven by calcium signaling pathways. When neurons release glutamate, it triggers the release of ATP from astrocytes, which subsequently activates the inositol trisphosphate (IP3) receptors in pericytes. This activation leads to the release of calcium from the endoplasmic reticulum, followed by the phosphorylation of myosin light chain kinase, resulting in pericyte relaxation. In addition, pericytes possess Piezo1 channels, which are mechanosensitive and can detect changes in blood flow shear stress, triggering calcium influx to regulate vascular tone (Hillman 2014). When neurons are excited, the release of glutamate activates the metabotropic receptors on the end‐feet of astrocytes, triggering the arachidonic acid metabolic pathway (such as the cyclooxygenase‐2/PGE2 pathway), which leads to the dilation of precapillary sphincters and results in functional hyperemia (with an increase in blood flow of up to 30%–50%) (Iadecola 2004). It is important to note that this regulation is bidirectional: Chronic hypoperfusion can promote leukocyte infiltration through the expression of ICAM‐1 in endothelial cells, exacerbating neuronal mitochondrial dysfunction and forming a vicious cycle (Zonta et al. 2003). The latest single‐cell sequencing studies have revealed that the KATP channels specifically expressed by pericytes are the key molecular switches for the timely regulation of NVC (Iadecola 2004). The structural basis for its functional implementation is the highly specialized NVU, in which the end‐feet of astrocytes cover approximately 90% of the brain capillary surface, forming the anatomical bridge for neurovascular signal transmission (Iadecola 2004). At the capillary level, pericytes utilize α‐smooth muscle actin and the RhoA/ROCK signaling pathways to fine‐tune capillary resistance during blood pressure fluctuations, thereby preventing damage caused by hyperperfusion.

Hypoxic conditions induce the production of mitochondrial reactive oxygen species in pericytes, which subsequently release adenosine to dilate blood vessels, thereby complementing arterial regulation both temporally and spatially (Magaki et al. 2019). The dynamic interaction between pericytes and the BBB is facilitated by their structural role: Pericytes cover 30%–70% of the microvascular surface and maintain BBB integrity through tight junctions with endothelial cells via N‐cadherin (Kaplan et al. 2020). Moreover, pericytes regulate BBB function by secreting Ang‐1/Tie2 signaling molecules, which inhibit vesicular transport in endothelial cells, thereby reducing leakage (Hillman 2014).

### 4.2 Dual‐Edged Roles of NVC in Neurodegenerative Diseases

NVC is intricately linked to AD through several shared pathomechanisms, including endothelial damage and BBB breakdown, which contribute to neurodegeneration and are implicated in nearly 90% of dementia cases (Vollhardt et al. 2025). Impairment in NVC is a key event in AD progression, with alterations in G protein‐coupled receptors exacerbating these effects, suggesting a potential therapeutic target to address brain blood flow and memory impairments. Decreased NVC is associated with structural brain changes in AD, particularly linked to white matter hyperintensities and lobar intracerebral hemorrhage, indicating early vascular damage in the disease process (van Dijk et al. 2025). Reduced cerebral blood flow, associated with the APOE4 genotype and sedentary behavior, negatively impacts NVC in AD, with exercise showing improvements in neurovascular function, particularly in APOE4 carriers (Anderle et al. 2025). Microglia regulate cerebral blood flow and NVC through the CD39‐initiated breakdown of extracellular ATP, suggesting a potential link between these processes in AD (Fu et al. 2025). Neurovascular aging impairs brain function by disrupting oxygen and glucose delivery, which is particularly detrimental in AD (Santisteban and Iadecola 2025). In addition, a negative correlation between β‐amyloid accumulation and cerebral blood flow response to neuronal activation indicates that higher β‐amyloid levels are associated with impaired cerebrovascular function, representing an early pathological change in AD (Vestergaard et al. 2025). Cerebrovascular dysfunction, including impaired NVC, is implicated in AD pathogenesis, as endothelial dysfunction and chronic vascular inflammation may promote amyloid pathology and accelerate neurodegenerative processes (Fekete et al. 2025).

The interaction between PD and NVC is complex and multifaceted. Cerebrovascular dysfunction in PD, characterized by increased latency in specific brain regions, suggests a significant role of NVC in its pathophysiology, although strong clinical associations are lacking (van der Horn et al. 2024). Impaired NVC in PD is evidenced by decreased global CBF and altered coupling in the parieto‐occipital and temporo‐occipital cortices, which are linked to motor and non‐motor impairments, enhancing the understanding of PD's pathophysiological mechanisms (Li, Liu, et al. 2023). The NVU's impairment, associated with neurodegenerative diseases like PD, highlights aging's role in increasing susceptibility to such conditions (Liu et al. 2023). Rehabilitation in PD is linked to changes in NVC, with reduced CBF‐fALFF correlations and specific regional brain activity changes correlating with motor improvement, indicating brain reorganization and compensation (Li, Wang, et al. 2023). Furthermore, PD patients exhibit abnormal NVC, with levodopa therapy improving NVC by modulating neuronal activity, reflecting a complex interplay between dopaminergic and nondopaminergic systems (Wu et al. 2024).

HD, a neurodegenerative disorder characterized by progressive motor, cognitive, and psychiatric impairments, is associated with altered NVC, as evidenced by changes in fMRI responses, which include reduced signals in some brain regions and increased signals in others, indicating potential differences in hemodynamics in HD patients (Clark et al. 2002). Impaired NVC, along with endothelial dysfunction, plays a significant role in the pathogenesis of HD, suggesting that viewing HD as a neurovascular disorder could lead to more effective treatment strategies (Hu et al. 2025). In the zQ175DN mouse model of HD, the accumulation of mutant huntingtin protein in astrocytes and pericytes, which are crucial for NVC, correlates with reduced synchronous brain activity and altered functional brain dynamics, highlighting a potential link between neurovascular dysfunction and disease progression (Vasilkovska, Verschuuren, et al. 2024). In addition, alterations in NVC in HD have been associated with impaired CBF and cerebrovascular reactivity, with early cortical hyperperfusion observed at 3 months of age, followed by reduced CBF and CVR impairments by 15 months, suggesting that these vascular changes may contribute to neurodegeneration in HD (Vasilkovska, Salajeghe, et al. 2024).

In summary, NVC occupies a pivotal yet paradoxical role in neurodegenerative diseases, serving as a critical bridge between basic mechanisms and clinical applications. Its dysfunction is a shared pathomechanism across disorders such as AD, PD, and HD. This central position in disease pathogenesis underscores its “double‐edged sword” effect, contributing to progression while simultaneously revealing vulnerable nodes for intervention. Consequently, targeting the NVU presents a promising therapeutic avenue for mitigating disease progression.

### 4.3 Multimodal Imaging in NVC: Bench‐to‐Bedside

The integration of TCD, fMRI, and fNIRS has emerged as a major research focus in neuroscience. Each modality provides unique advantages—TCD offers high temporal resolution, fMRI provides superior spatial resolution (Logothetis 2008), and fNIRS is valued for its convenience and non‐invasiveness. Combined, these techniques enable a comprehensive assessment of hemodynamic responses associated with neural activity, enhancing the precision of NVC measurements critical to both basic and clinical neuroscience. This multimodal approach is particularly valuable for real‐time brain function monitoring, including applications in brain–computer interfaces (Liao et al. 2013). By capturing dynamic changes in CBF and neural activity, researchers can translate these signals into external device control, advancing neurorehabilitation for patients with brain injuries and neurological disorders. Integrating NVC measurements with functional connectivity analysis offers deeper insights into how brain networks interact under various conditions. The joint use of fMRI, TCD, and fNIRS facilitates detailed mapping of the relationship between hemodynamics and neural activity, elucidating mechanisms underlying functional connectivity alterations in neurological diseases (Drew 2022).

Developing accurate computational models of NVC has become a central focus in the field. By combining multimodal imaging data with biophysical simulations, researchers can model complex neurovascular interactions, revealing how vascular dysfunction contributes to neurodegenerative processes such as AD (Zehendner et al. 2014). Such models not only improve understanding of normal and pathological brain hemodynamics but also support the development of early diagnostic biomarkers and therapeutic strategies (Nunes et al. 2021).

Clinically, the combined application of TCD, fMRI, and fNIRS represents a growing trend (Chiarelli et al. 2017). This integration improves the accuracy of brain function assessment in conditions such as AD, stroke, and other neurodegenerative disorders by providing complementary data on CBF and neuronal activation (Burma et al. 2024). Consequently, multimodal imaging enhances clinical decision‐making and the development of personalized treatment plans.

Recent advances such as two‐photon microscopy have revolutionized NVC research, transitioning from in vivo imaging to single‐cell analyses. Real‐time visualization of pericyte calcium signaling in transgenic mouse models (e.g., PDGFRβ‐CreERT2 with GCaMP indicators) has provided new insights into microvascular regulation (Meyer‐Baese et al. 2025). Furthermore, laser speckle contrast imaging studies demonstrate that pericytes regulate capillary blood flow redistribution with threefold greater efficiency than arterioles (Bonilauri et al. 2020)

Overall, the convergence of multimodal neuroimaging and advanced computational modeling is transforming both neuroscience and clinical medicine. Integrating CBF and metabolic imaging data promises to deepen our understanding of NVC mechanisms and support early, individualized diagnosis and treatment of neurological disorders (Nunes et al. 2021).

### 4.4 Future Direction

In the future, NVC research will continue to evolve towards interdisciplinary collaboration and clinical translation. By integrating advanced technologies such as artificial intelligence and multi‐omics analysis, in‐depth exploration of the mechanisms of NVC in health and disease will bring new breakthroughs to the field of neuroscience and brain health (Attwell and Iadecola 2002; Iadecola 2017). Moreover, as NVC research involves multiple disciplines including neuroscience, molecular biology, imaging, and clinical medicine, further strengthening of interdisciplinary collaboration and integration of multi‐omics data with high‐resolution imaging techniques will be necessary to uncover the complex mechanisms underlying NVC.

### 4.5 Limitations

Our study provides a pioneering systematic analysis of the research landscape using bibliometric methods, enabling a quantitative evaluation of the field's evolution. By leveraging tools such as VOSviewer and CiteSpace, the analysis offers an objective and reproducible assessment of key contributors, thematic trends, and knowledge gaps, supported by an extensive review of English‐language literature from the WoSCC. This approach establishes a robust framework to guide future investigations. However, several limitations must be acknowledged. First, reliance on WoSCC‐exclusive, English‐language articles introduces potential selection bias, as relevant studies from databases like PubMed, Scopus, or non‐English sources may have been excluded. Second, inconsistencies in author/institutional nomenclature and publication delays may affect analytical accuracy and timeliness. Third, while NVC research holds significant translational potential, critical technical challenges persist. Current imaging modalities (e.g., MRI, PET) lack the spatiotemporal resolution to comprehensively capture cerebral hemodynamic and neural activity dynamics. Similarly, pharmacological interventions targeting NVC—such as angiogenesis promotion, neuronal repair, or anti‐inflammatory therapies in stroke models—remain exploratory, underscoring the need for mechanistic validation and clinical optimization.

## 5 Conclusion

This study maps three decades of progress in NVC, emphasizing global collaboration and evolving priorities. Future efforts must prioritize molecular mechanisms and multimodal imaging integration, and hemodynamic‐metabolic coupling to unlock biomarkers for early diagnosis and personalized therapies in neurological disorders.

## Author Contributions

All authors' contributions are equal. All authors have read and agreed to the published version of the manuscript.

## Funding

This work was supported by Shanxi Health Committee Foundation Project (2024057) and Fundamental Research Program of Shanxi Province (202403021222432).

## Consent

The authors have nothing to report.

## Conflicts of Interest

The authors declare no conflicts of interest.

## Supporting information




**Supplementary Tables**: brb371058‐sup‐0001‐SuppMat.docx

## Data Availability

The authors in the article/supplementary material will provide the original data that support this paper's conclusions. Further inquiries can be directed to the corresponding authors.
