# Supplementary material for: Neurovascular Coupling: Scientometric Analysis of 30 Years Research (1996–2025)
Source: Brain Behav. 2025 Nov 11;15(11):e71058. doi: 10.1002/brb3.71058 (PMC12606022; doi:10.1002/brb3.71058)
Supplement: Supplementary file 1 — Supplementary Tables: brb371058‐sup‐0001‐SuppMat.docx [file BRB3-15-e71058-s001.docx]

**Neurovascular Coupling: Scientometric Analysis of 30 Years Research(1996-2025)**

Running title: Neurovascular Coupling

**Supplemental Table S1. Main Information based on Bibliometrix**

| **Description** | **Results** |
| --- | --- |
| Timespan | 1996:2025 |
| Sources (Journals, Books, etc) | 484 |
| Documents | 2047 |
| Document Average Age | 8.27 |
| Average citations per doc | 42.37 |
| References | 80592 |
| DOCUMENT CONTENTS |  |
| Keywords Plus (ID) | 4761 |
| Author's Keywords (DE) | 3842 |
| AUTHORS |  |
| Authors | 8494 |
| Authors of single-authored docs | 57 |
| AUTHORS COLLABORATION |  |
| Single-authored docs | 59 |
| Co-Authors per Doc | 6.22 |
| International co-authorships % | 31.12 |
| DOCUMENT TYPES |  |
| article | 1720 |
| review | 327 |

**Supplementary Table S2.** Most Relevant Countries by Corresponding Author Contributions based on Bibliometrix

| Rank | **Country** | **Articles** | **Articles %** | **SCP** | **MCP** | **MCP %** |
| --- | --- | --- | --- | --- | --- | --- |
| 1 | USA | 663 | 32.4 | 498 | 165 | 24.9 |
| 2 | CHINA | 195 | 9.5 | 156 | 39 | 20 |
| 3 | GERMANY | 194 | 9.5 | 118 | 76 | 39.2 |
| 4 | UNITED KINGDOM | 168 | 8.2 | 116 | 52 | 31 |
| 5 | CANADA | 160 | 7.8 | 108 | 52 | 32.5 |
| 6 | JAPAN | 89 | 4.3 | 75 | 14 | 15.7 |
| 7 | FRANCE | 74 | 3.6 | 44 | 30 | 40.5 |
| 8 | ITALY | 63 | 3.1 | 43 | 20 | 31.7 |
| 9 | DENMARK | 50 | 2.4 | 30 | 20 | 40 |
| 10 | SWITZERLAND | 48 | 2.3 | 23 | 25 | 52.1 |

**Supplementary Table S3.** Most_Relevant_Affiliations_based on Bibliometrix

| **Rank** | **Affiliation** | **Articles** |
| --- | --- | --- |
| 1 | HARVARD UNIVERSITY | 220 |
| 2 | UNIVERSITY OF CALIFORNIA SYSTEM | 165 |
| 3 | UNIVERSITY OF OKLAHOMA SYSTEM | 158 |
| 4 | UNIVERSITY OF OKLAHOMA HEALTH SCIENCES CENTER | 153 |
| 5 | UNIVERSITY OF CALGARY | 147 |
| 6 | PENNSYLVANIA COMMONWEALTH SYSTEM OF HIGHER EDUCATION (PCSHE) | 138 |
| 7 | UNIVERSITY OF COPENHAGEN | 120 |
| 8 | HUMBOLDT UNIVERSITY OF BERLIN | 118 |
| 9 | BERLIN INSTITUTE OF HEALTH | 115 |
| 10 | FREE UNIVERSITY OF BERLIN | 114 |
